# Identification of novel cis-regulatory elements of *Eya1* in *Xenopus laevis* using BAC recombineering

**DOI:** 10.1038/s41598-017-15153-7

**Published:** 2017-11-03

**Authors:** Santosh Kumar Maharana, Nicolas Pollet, Gerhard Schlosser

**Affiliations:** 1 School of Natural Sciences and Regenerative Medicine Institute (REMEDI), National University of Ireland, Galway, Biosciences Research Building, Newcastle Road, Galway, Ireland; 2 Evolution, Génomes, Comportement & Ecologie, CNRS, IRD, Univ. Paris-Sud, Université Paris-Saclay, 91198 Gif-sur-Yvette, France; 3 Present Address: New York University, College of Dentistry, Department of Basic Science and Craniofacial Biology, New York, USA

## Abstract

The multifunctional Eya1 protein plays important roles during the development of cranial sensory organs and ganglia, kidneys, hypaxial muscles and several other organs in vertebrates. Eya1 is encoded by a complex locus with candidate cis-regulatory elements distributed over a 329 kbp wide genomic region in *Xenopus*. Consequently, very little is currently known about how expression of Eya1 is controlled by upstream regulators. Here we use a library of *Xenopus tropicalis* genomic sequences in bacterial artificial chromosomes (BAC) to analyze the genomic region surrounding the *Eya1* locus for enhancer activity. We used BAC recombineering to first create GFP reporter constructs, which were analysed for enhancer activity by injection into *Xenopus laevis* embryos. We then used a second round of BAC recombineering to create deletion constructs of these BAC reporters to localize enhancer activity more precisely. This double recombineering approach allowed us to probe a large genomic region for enhancer activity without assumptions on sequence conservation. Using this approach we were able to identify two novel cis-regulatory regions, which direct Eya1 expression to the somites, pharyngeal pouches, the preplacodal ectoderm (the common precursor region of many cranial sensory organs and ganglia), and other ectodermal domains.

## Introduction

Eya1 is a multifunctional protein that acts as a transcriptional coactivator of the transcription factor Six1 and has additional functions in the cytoplasm, where it affects cell polarity and promotes asymmetric cell divisions, partly mediated by its phosphatase activity^1,2^. Together, Eya1 and Six1 play multiple important roles during the development of sensory organs and ganglia derived from cranial placodes in vertebrates such as the inner ear and the lateral line system. In addition, they have been implicated in the development of kidneys, hypaxial muscles and several other organs.

In humans, mutations in either *Eya1* or *Six1* result in branchio-oto-renal (BOR) or branchio-otic (BO) syndrome with congenital hearing deficits^3^. Mutations or knockdown of these genes in mice, zebrafish and *Xenopus* have shown their central importance for cranial placode development, where they are required both for the proliferation of progenitors of placodal neurons and sensory cells and for their subsequent differentiation^4–11^.

During vertebrate embryonic development, Eya1 is first expressed at the beginning of gastrulation and becomes upregulated in a horseshoe shaped region of the ectoderm surrounding the anterior neural plate at the end of gastrulation, where it is coexpressed with Six1^12–17^. This expression domain, the preplacodal region (PPR) delineates a panplacodal primordium that will subsequently give rise to all cranial placodes^18–21^. With exception of the lens placode, all placodes maintain expression of Eya1 and Six1 at least throughout tailbud stages, when additional expression domains in the pharyngeal pouches, hypaxial muscles and kidney primordia appear^12–17^.

Despite the central importance of *Eya1* for cranial placode development, very little is known about its upstream regulation. Recent studies implicated FGFs, BMP inhibitors, and Wnt inhibitors derived from the neural plate and the underlying mesoderm in PPR induction^22–24^. Other studies demonstrated that only non-neural ectoderm is competent to express Eya1 and Six1 in response to inducing signals and showed that Dlx3/5, GATA2/3, FoxI, AP2, and Msx1/2 are among the transcription factors required for non-neural competence^25–27^.

However, how these various inputs are integrated at the level of cis-regulatory sequences is not well understood. The *Eya1* locus consists of many exons (19 exons in *Xenopus tropicalis* with the coding region extending from exon 1 to 19) spanning a large chromosomal region (approx. 60 kbp of chromosome 6 in *X. tropicalis*) and separated by extensive regions of non-coding DNA from its 5′ neighbor *MSC* (146 kbp in *X. tropicalis*) and its 3′ neighbor *XKR9* (124 kbp in *X. tropicalis*). A previous study has identified 29 conserved noncoding sequences (CNS) in this genomic region using sequence comparison between several vertebrate genomes (phylogenetic footprinting)^28^. Using electroporation of reporter constructs into chick embryos, ten of these CNS exhibited enhancer activity mimicking some of the known Eya1 expression domains^28^. However other Eya1 expression domains, in particular in the PPR, were not driven by any of the CNSs analyzed suggesting the existence of additional enhancers. This is not surprising, since many enhancers have been shown to maintain regulatory specificity while undergoing rapid sequence evolution and such enhancers will not be identifiable as CNS^29,30^.

While the recent recognition of enhancer-typical epigenetic signatures has provided us with additional tools to identify active enhancers by particular chromatin marks (e.g. presence of H3K27ac and absence of H3K27me3 marks) from ChIP-Seq data^31,32^, the predictive value of these methods is limited and prone to overlook enhancers with atypical epigenetic properties^33^. Ultimately, therefore, nonbiased approaches will be required to comprehensively identify cis-regulatory elements driving expression of a particular gene.

**Figure 1:**
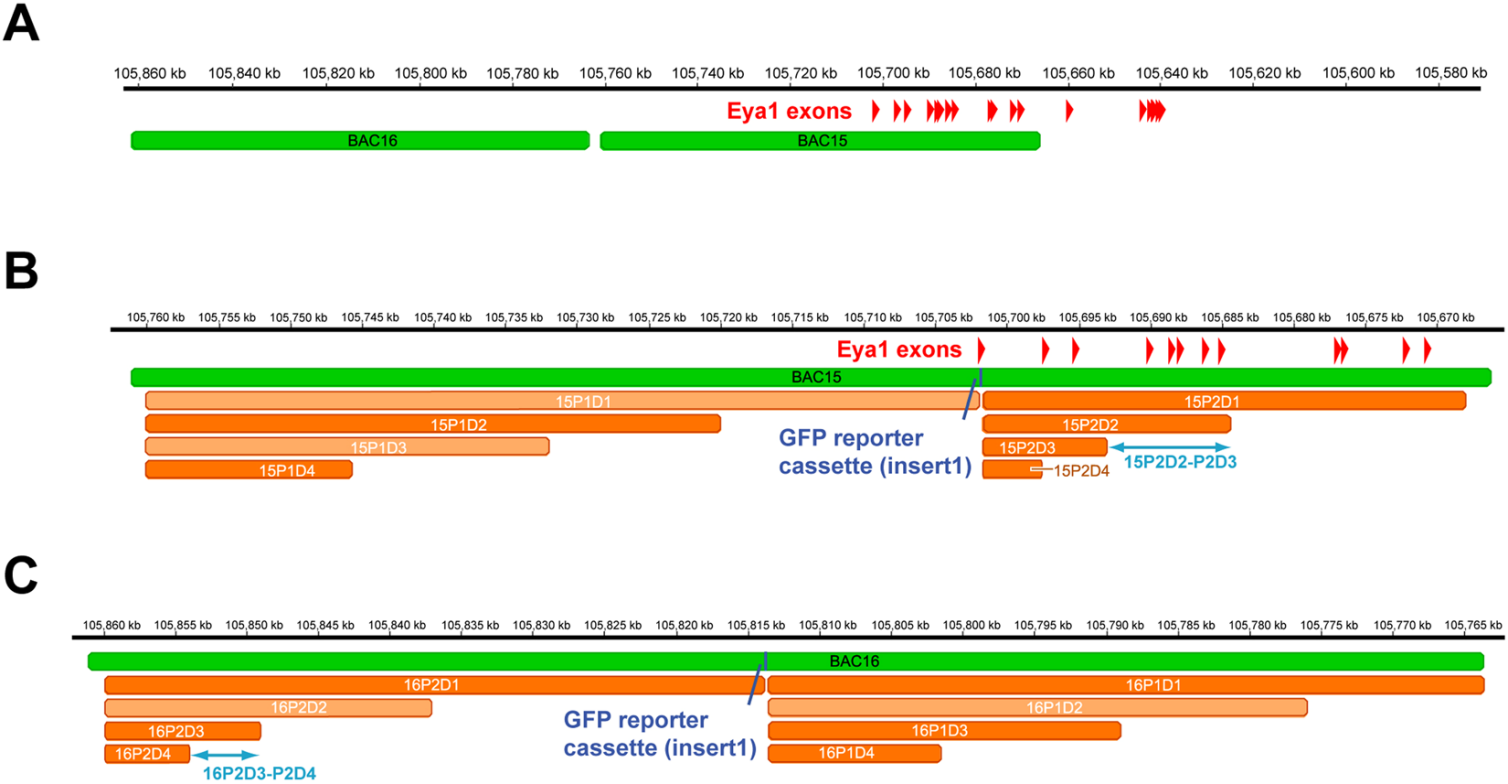
Genomic region of *Eya1* in *Xenopus tropicalis*. (**A**) Position of BACS 15 and 16 on chromosome 6 of *X.tropicalis*. Note that the precise position of the plus end (right side) for BAC16 has not been reported (see Suppl. Figure 1 and Table 1). (**B,C**) Detail of BAC15 (**B**) and BAC16 (**C**) with the positions of the GFP reporter cassette (insert 1, blue) and the regions deleted in various deletion constructs (orange) indicated. Deletion constructs depicted by faint orange color were not tested. Scale shows position on chromosome 6 of the *Xenopus tropicalis* genome (version 9.0; http://www.xenbase.org/). Blue double arrows show position of the two genomic regions with enhancer activity identified in the present study, BAC15P2D2-P2D3 and BAC16P2D3-P2D4.

**Figure 2:**
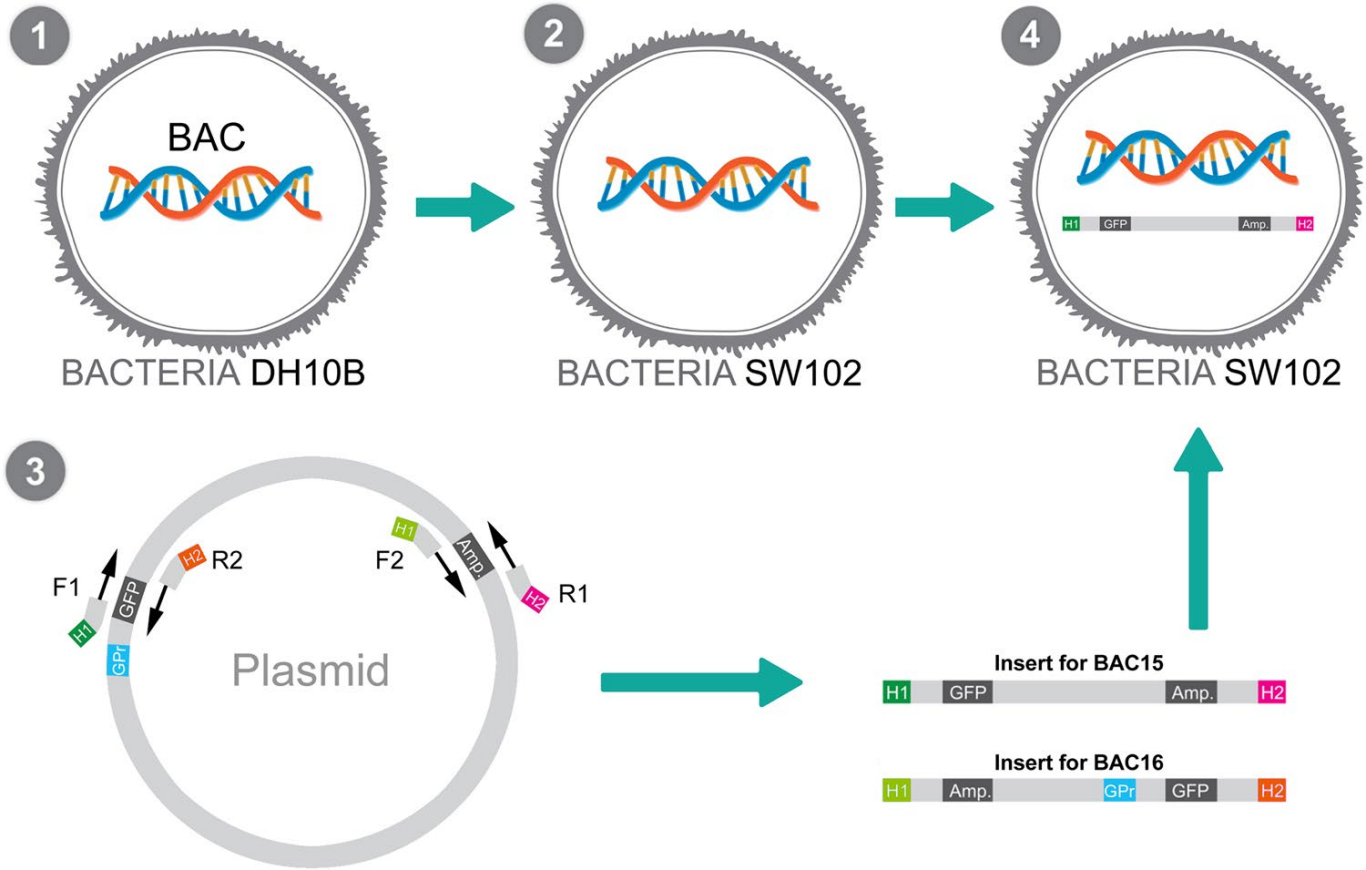
Overview of procedure for generating BAC15 and BAC16 reporter constructs. BACs were obtained from a BAC library maintained in the DH10B strain of *E. coli* (1). BACs were then purified and transformed into strain SW102 (or EL250; not shown) competent for recombineering (2). Primers with overhangs complementary to the targeted BAC region (homology arms H1 and H2) were used to amplify a cassette containing the *GFP* reporter and an ampicillin resistance gene (*Amp*) from plasmid pIS-GATA2-GFP using PCR. The insert amplified with primers F1 and R1 was used for BAC15 and did not contain a promoter, whereas the insert amplified with primers F2, R2 was used for BAC16 and contained the GATA2 minimal promoter (3).The insert was transformed into SW102 bacteria containing the appropriate BAC to allow for recombineering (4).

**Figure 3:**
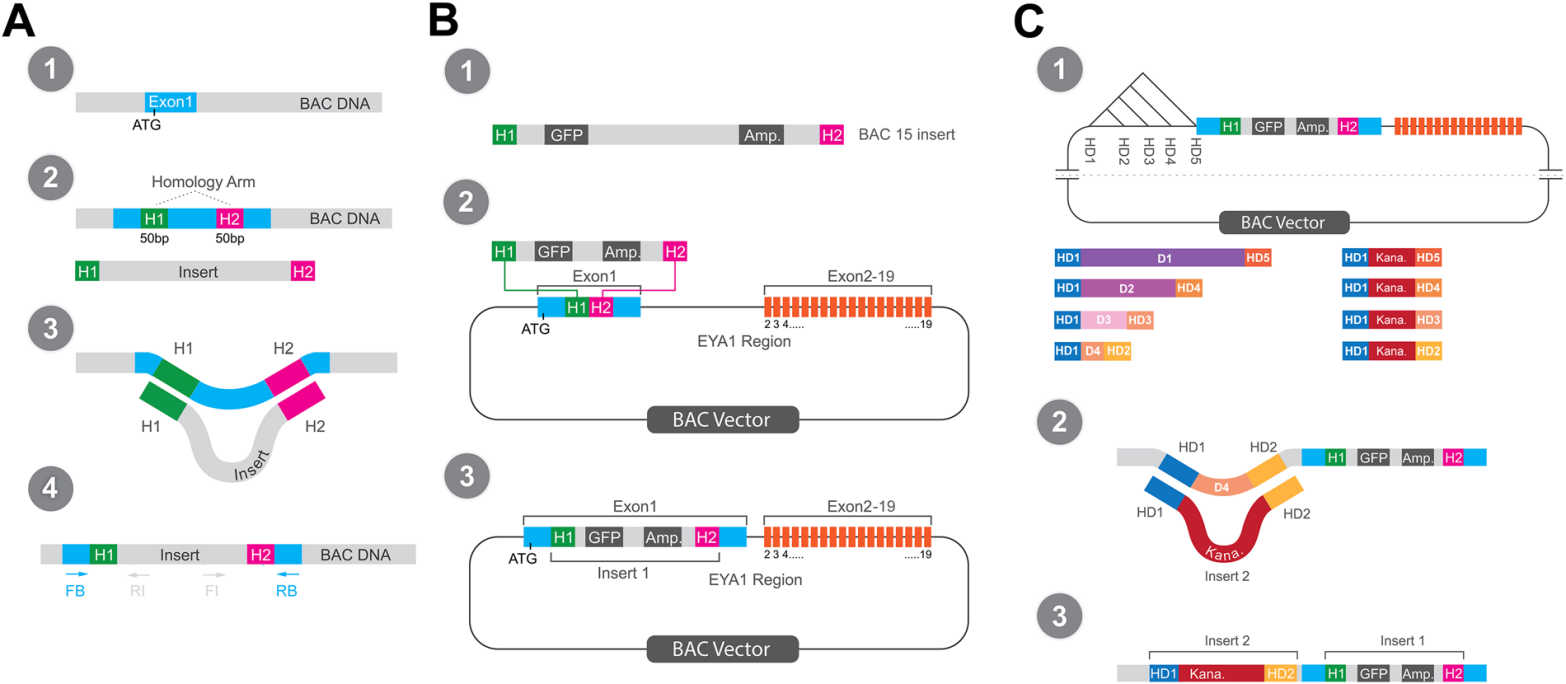
Double recombineering strategy to localize enhancers in large genomic regions. (**A**) Overview of recombineering procedure. A target region for insertion is selected in the BAC, for example the first coding exon of the gene of interest containing the start codon (ATG) (1). An insert (containing an antibiotic resistance gene and possibly other elements such as a reporter gene) with appropriate homology arms (H1, H2) complementary to BAC target sequences is transformed into bacteria containing the BAC (2). The insert is inserted into the BAC by homologous recombination (recombineering) replacing the original BAC sequence between H1 and H2 (3). Proper insertion is verified by PCR using forward (FB) and reverse primers (RB) for BAC sequences flanking the insertion, while proper orientation is verified by PCR combining FB with a reverse primer for an insert specific sequence (RI) or a forward primer for an insert specific sequence with RB (4). (**B**) Creation of reporter BACs in the first recombineering step. Insert 1 (here the insert for BAC15) contains an ampicillin resistance gene and a *GFP* reporter gene (1). Homology arms are designed to target the insert to a position immediately after the start codon in the first coding exon of *Eya1* during recombineering (2). BAC reporter construct after recombineering (3). (**C**) Creation of deletion constructs of BAC reporters in the second recombineering step. Sequences flanking large noncoding regions in the BAC are selected as new homology arms (HD1-HD5). Chosing different combinations of these for the insert 2 (containing a kanamycin resistance gene) will result in deletions of different extent (1). During recombineering the sequence D4 on the BAC between HD1 and HD2 (or between other homology arms chosen) will be deleted and replaced by the kanamycin resistance cassette (2). BAC reporter deletion construct after second step of recombineering containing insert 1 and 2 (3).

Here we use a library of *Xenopus tropicalis* genomic sequences in bacterial artificial chromosomes (BAC)^34^ to test the genomic region surrounding the *Eya1* locus on chromosome 6 for enhancer activity (this region corresponds to two genomic regions in the pseudo-tetraploid species *Xenopus laevis* represented by scaffold 52 and chr.6 S in the *X. laevis* assembly v9.1). Each BAC contains a large (70–130 kbp) piece of genomic DNA so that a few BACs cover the entire genomic region of interest (Fig. 1, Suppl. Figure 1). We used BAC recombineering (Figs 2 and 3) to first create GFP reporter constructs, which could be analysed for enhancer activity by injection into *Xenopus laevis* embryos^35,36^. We then used a second round of BAC recombineering to create deletion constructs of these BAC reporters to localize enhancer activity more precisely (Fig. 3). Using this approach we were able to probe large Eya1 genomic regions for enhancer activity and to identify two regions (9 and 5.5 kbp long, respectively), which direct Eya1 expression to the PPR and additional domains.

## Results

The *X. tropicalis* BAC library contains several BACs covering the region between the *Eya1* locus and its neighboring genes *MSCI* and *XKR9* (Suppl. Table 1, Suppl. Figure 1). We selected BAC15 and BAC16 for more detailed analysis of enhancer activity because they cover the 5′ end of the *Eya1* coding region and upstream sequences (Fig. 1). In a first round of BAC recombineering we inserted a cassette containing a *GFP* reporter gene and an ampicillin resistance gene into BAC 15 and 16, thereby creating BAC GFP reporter constructs (Figs 2 and 3A,B). For BAC15, which contains the first coding exon of Eya1, we inserted GFP immediately past the start codon of *Eya1*, where it is under control of the *Eya1* promoter. For BAC16, which does not overlap with the *Eya1* coding region, we inserted a GFP cassette preceded by the GATA2 minimal promoter into a central position of the BAC. Proper insertion and orientation of the insert was confirmed by PCR and sequencing. BACs were injected into fertilized eggs of *Xenopus laevis* before the first cell division.

### General observations on BAC injected embryos

In contrast to plasmids, which degrade quickly and become distributed in a mosaic fashion, injected BACs have been reported to replicate in the developing embryo with BAC encoded cis-regulatory regions driving reporter gene expression in the appropriate endogenous domains^35,37^. We confirm this here for the full length BAC15 and BAC16 reporter constructs (BAC15-FL, BAC16-FL) each of which drives GFP expression in some of the endogenous domains of Eya1 (see below). Reporter gene expression was typically observed in the majority of embryos injected with the BAC reporters (Tables 1 and 2). Control embryos injected with unrecombineered BAC15 or BAC16 lacking the reporter insert, did not show any GFP expression (Tables 1 and 2; Suppl. Figure 2).

**Table 1:** Expression domains mediated by BAC15.

Expression Constructs	Neural plate stage embryos (14–18)			Tailbud stage embryos (23–40)			
	Epidermis % (n)	PPE % (n)	Neural plate % (n)	Epidermis % (n)	Head ectoderm/ placodes % (n)	Somites % (n)	Pharyngeal pouches/gut % (n)
BAC15-control^1^	0 (53)	0 (53)	0 (53)	0 (8)	0 (8)	0 (8)	0 (8)
BAC15-FL^2^	81 (124)	75 (124)	81 (124)	74 (114)	74 (114)	71 (114)	30 (114)
BAC15-P1D1^3^	nd	nd	nd	nd	nd	nd	nd
BAC15-P1D2^3^	78 (49)	73 (49)	78 (49)	76 (129)	62 (129)	75 (129)	33 (129)
BAC15-P1D3^3^	nd	nd	nd	nd	nd	nd	nd
BAC15-P1D4^3^	79 (43)	72 (43)	79 (43)	90 (10)	90 (10)	90 (10)	60 (10)
BAC15-P2D1^3^	0 (46)	0 (46)	0 (46)	0 (64)	0 (64)	3 (64)	0 (64)
BAC15-P2D2^3^	0 (44)	0 (44)	0 (44)	0 (61)	0 (61)	0 (61)	0 (61)
BAC15-P2D3^3^	97 (37)	97 (37)	89 (37)	50 (4)	50 (4)	25 (4)	0 (4)
BAC15-P2D4^3^	100 (29)	97 (29)	93 (29)	67 (6)	67 (6)	50 (6)	0 (6)

n: number of embryos analyzed; nd: not determined.

^1^Empty BAC15 (no insert).

^2^BAC15 full length (FL).

^3^BAC15 deletion constructs (see Fig. 3).

**Table 2:** Expression domains mediated by BAC16.

Expression Constructs	Neural plate stage embryos (14–18)			Tailbud stage embryos (23–40)			
	Epidermis % (n)	PPE % (n)	Neural plate % (n)	Epidermis % (n)	Head ectoderm/ placodes % (n)	Somites % (n)	Pharyngeal pouches % (n)
BAC16-control^1^	0 (59)	0 (59)	0 (59)	0 (8)	0 (8)	0 (8)	0 (8)
BAC16-FL^2^	71 (234)	67 (234)	68 (234)	60 (148)	76 (148)	35 (148)	0 (50)
BAC16-P1D1^3^	85 (54)	83 (54)	78 (54)	78 (124)	69 (124)	23 (124)	0 (82)
BAC16-P1D2^3^	nd	nd	nd	nd	nd	nd	nd
BAC16-P1D3^3^	55 (29)	41 (29)	41 (29)	65 (69)	46 (69)	26 (69)	0 (69)
BAC16-P1D4^3^	72 (152)	68 (152)	71 (152)	73 (146)	60 (146)	50 (146)	0 (146)
BAC16-P2D1^3^	7 (41)	0 (41)	0 (41)	0 (51)	0 (51)	2 (51)	0 (51)
BAC16-P2D2^3^	nd	nd	nd	nd	nd	nd	nd
BAC16-P2D3^3^	0 (48)	0 (48)	0 (48)	0 (73)	0 (73)	0 (73)	0 (73)
BAC16-P2D4^3^	69 (36)	50 (36)	44 (36)	61 (70)	49 (70)	33 (70)	16 (70)

n: number of embryos analyzed; nd: not determined.

^1^Empty BAC16 (no insert).

^2^BAC16 full length (FL).

^3^BAC16 deletion constructs (see Fig. 3).

However, in contrast to transgenic *Xenopus* embryos, in which candidate regulatory regions were integrated into the genome by restriction enzymes or SceI meganuclease^38,39^, embryos injected with BAC reporter constructs only rarely show completely homogeneous reporter expression in endogenous domains and more often exhibit some degree of mosaicism with only subsets of cells expressing the reporter. In many embryos only unilateral expression was observed, presumably reflecting unequal partitioning of the injected BACs to the first two blastomeres. While reporter gene activity was evident as GFP fluorescence in living embryos, the signal was sometimes weak or difficult to distinguish from background autofluorescence. We therefore analyzed embryos by *in situ* hybridization for *GFP*, which allowed the detection of weaker signals and was less prone to background staining.

### BAC15 reporter gene expression

**Figure 4:**
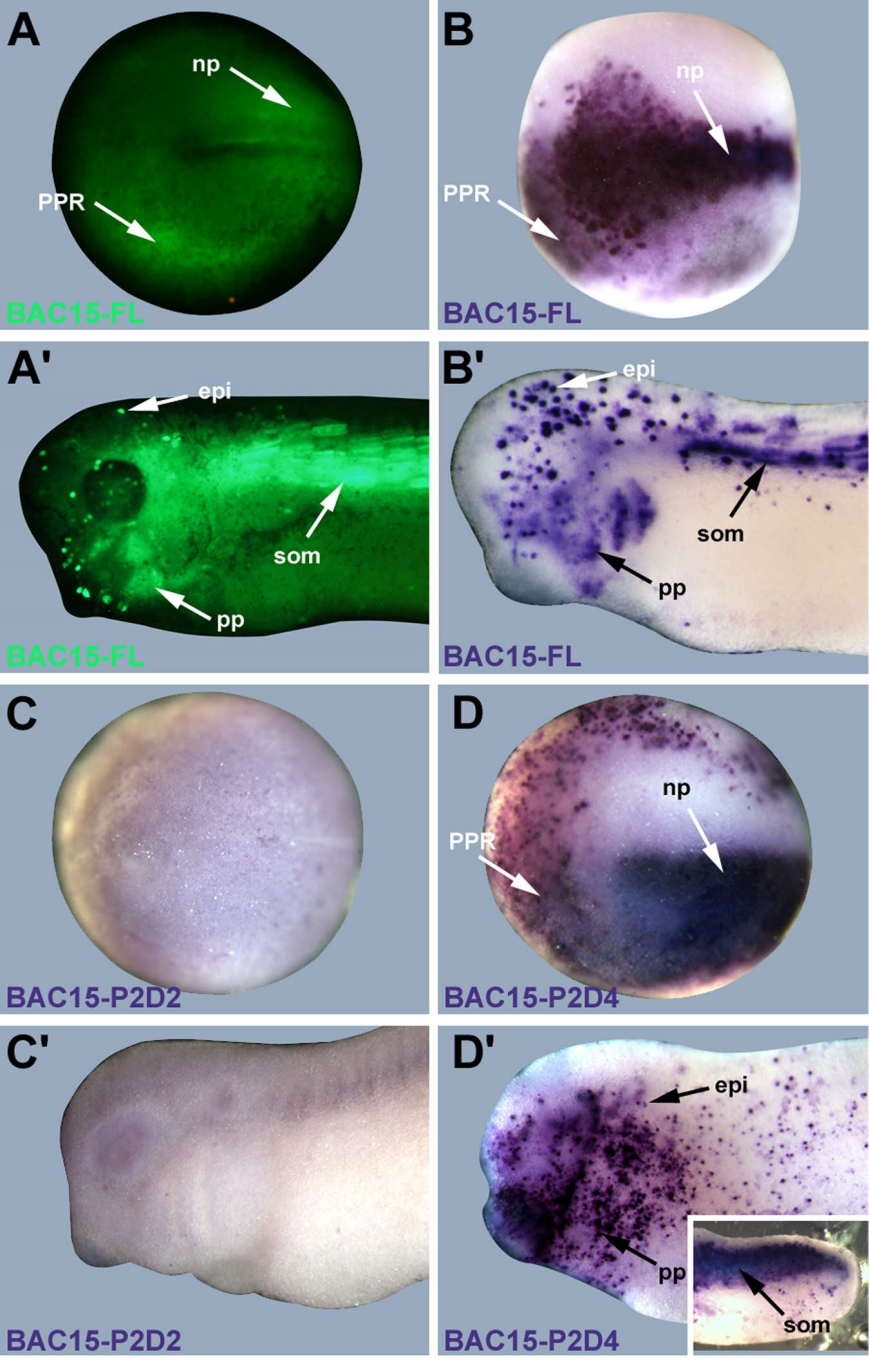
GFP reporter expression driven by BAC15. Embryos of *Xenopus laevis* at neural plate (**A**–**D**: dorsal view, anterior to the left) and tailbud stages (**A**’–**D**’: lateral view, anterior to the left) after injection with BAC15 reporter constructs at the one-cell stage. **A,A’,B,B’**: GFP expression after injection of full length BAC15 reporter (BAC15-FL) in living *Xenopus* embryos (**A**,**A**’) or as revealed by *GFP in situ* hybridization (B,B’). **C,C’**: Absence of *GFP* expression domains after injection of BAC15-P2D2 deletion construct. **D,D’**: *GFP* expression domains after injection of BAC15-P2D4 deletion construct recapitulate those observed after BAC15-FL injection. Insert in **D’** shows somitic expression in trunk region of a different embryo (tail end to the right). Abbreviations: epi: epidermis; np: neural plate; pp: pharyngeal pouches; PPR: preplacodal region; som: somites.

Embryos injected with full length BAC15 reporter constructs first showed GFP expression at the end of gastrulation in the PPR and the neural plate. Scattered epidermal cells were also GFP positive (Fig. 4A,B; Table 1). From neural fold stages on, GFP was strongly upregulated in the paraxial mesoderm and continued to be expressed in the somites at subsequent tailbud stages (Fig. 4A’,B’; Table 1). At tailbud stages, the pharyngeal pouches (and occasionally scattered cells in other parts of the gut) also began to express GFP and GFP expression persisted in the epidermis, but not in placodes or neural tube (Fig. 4A’,B’; Table 1).

This pattern of expression mimics Eya1 expression in the endomesoderm (somites and pharyngeal pouches). In the ectoderm it also reflects early expression of Eya1 in the PPR but not its subsequent expression in cranial placodes. Taken together this suggests the presence of endomesodermal and PPR enhancers but not of dedicated placodal enhancers in BAC15.

It should be noted though that reporter gene expression extends beyond endogenous expression of Eya1 in the ectoderm. While endogenous Eya1 is only weakly and transiently expressed in the neural plate and is mostly confined to the PPR at neural plate stages, we observe strong reporter gene expression not only in the PPR but also in the neural plate as well as in scattered epidermal cells. Moreover, epidermal reporter expression persists into tailbud stages, when endogenous Eya1 is largely confined to placodes. There are several possible explanations for these mismatched expression domains which will be briefly considered in the discussion below.

### Deletion analysis of BAC15

To localize the endodermal, somitic and PPR enhancers on BAC15 more precisely, we next deleted different parts of BAC15 in a second round of recombineering by replacing regions of various sizes with a selection cassette containing a kanamycin resistance gene (Fig. 3C). We designed two nested sets of deletion constructs, one on each side of the GFP reporter gene cassette which was left in place (Fig. 1). Again, proper insertion of the kanamycin cassette was confirmed by PCR and sequencing and BAC deletion constructs were injected into fertilized eggs of *Xenopus laevis* before the first cell division.

We first deleted a large region of BAC15 upstream of the Eya1 coding region (Fig. 1). Since our attempts to delete the entire upstream region (P1D1) were not successful, we started with a smaller deletion (P1D2). Injection of BAC15-P1D2 fully recapitulated all GFP expression domains observed after injection of the full length BAC15-FL reporter (Table 1) indicating that none of the enhancers is located in the deleted region P1D2. This was confirmed by injection of the smaller deletion construct P1D4 which also recapitulated all GFP domains of BAC15-FL (Table 1).

We next deleted a large region (P2D1) of BAC15 downstream of the reporter cassette in exon1 of Eya1 (Fig. 1). This deletion construct, BAC15-P2D1 was unable to drive GFP expression in any of the domains observed after BAC15-FL expression indicating that all enhancers are contained in the deleted region P2D1. The same result was obtained after injection of a construct with the smaller P2D2 deletion suggesting that enhancers are contained in the P2D2 region (Fig. 4C,C’; Table 1). However, all GFP domains driven by BAC15-FL except pharyngeal pouches were recovered after injection of constructs with the even smaller deletions P2D3 or P2D4 (Fig. 4D,D’; Table 1) indicating that all enhancers except possibly the enhancer for pharyngeal pouches are located in the approximately 9 kb domain covered by P2D2 but not P2D3 (BAC15P2D2-P2D3). However, since pharyngeal pouches were positive only in 30% of embryos injected with BAC15-FL and only relatively few embryos were analysed for BAC15-P2D3 and BAC15-P2D4, our data do not allow us to make conclusive statements about the presence or absence of a pharyngeal pouch enhancer in this domain.

### BAC16 reporter gene expression

**Figure 5:**
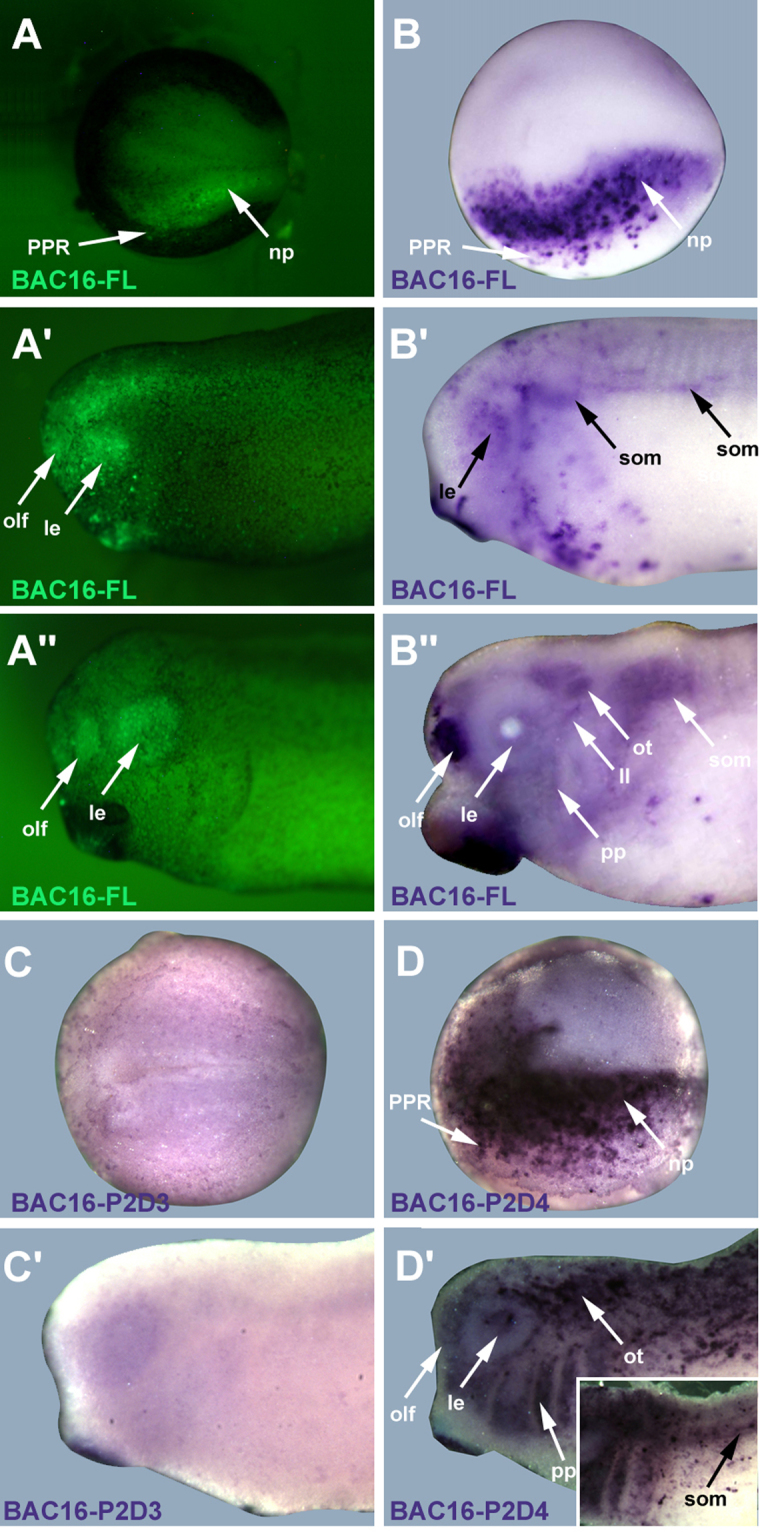
GFP reporter expression driven by BAC16. Embryos of *Xenopus laevis* at neural plate (**A**–**D**: dorsal view, anterior to the left) and tailbud stages (**A’**–**D’**, **A**”,**B**”: lateral view, anterior to the left) after injection with BAC16 reporter constructs at the one-cell stage. **A–A”, B–B”**: GFP expression after injection of full length BAC16 reporter (BAC16-FL) in living *Xenopus* embryos (**A**–**A**”) or as revealed by *GFP in situ* hybridization (B–B”). **C,C’**: Absence of *GFP* expression domains after injection of BAC16-P2D3 deletion construct. **D,D’**: *GFP* expression domains after injection of BAC16-P2D4 deletion construct recapitulate those observed after BAC16-FL injection. Insert in D’ shows somitic expression in trunk region of a different embryo (tail end to the right). Abbreviations: epi: epidermis; le: lens placode; ll: lateral line placodes; np: neural plate; olf: olfactory placode; ot: otic placode/vesicle; pp: pharyngeal pouches; PPR: preplacodal region; som: somites.

Embryos injected with full length BAC16 reporter constructs first showed GFP expression at the end of gastrulation in the PPR and the neural plate. Scattered epidermal cells were also GFP positive (Fig. 5A,B; Table 2). Expression domains driven by BAC16-FL at neural plate stages, thus, are very similar to those driven by BAC15-FL although there is almost no overlap between these two BACs. From neural fold stages on, GFP is seen in the paraxial mesoderm and at early tailbud stages in the somites although expression is often weaker and less extensive than after BAC15-FL injection (Fig. 5A’,B’; Table 2). At later tailbud stages, GFP continues to be expressed in scattered epidermal cells and cranial ectoderm including both epidermal cells and various cranial placodes, including the lens, profundal/trigeminal, olfactory, otic and lateral line placodes (Fig. 5A”,B”; Table 2). We were unable to determine whether GFP is also expressed in epibranchial placodes due to their small size and the partly mosaic GFP expression.

This pattern of expression mimics Eya1 expression in somites, the PPR and in the cranial placodes and suggests the presence of somite, PPR and later placode enhancers in BAC16. Thus, several of the enhancers located on BAC16 (e.g. for PPR and somites) appear to serve redundant functions to enhancers located on BAC15. Furthermore, similar to BAC15, BAC16 also drives ectodermal reporter gene expression that extends beyond endogenous expression of Eya1 in the early neural plate, the epidermis and the lens placode (which normally downregulates Eya1^16^).

### Deletion analysis of BAC16

To localize the somitic, PPR and placodal enhancers on BAC16 more precisely, we next created and confirmed two nested sets of deletion constructs for BAC16, one on each side of the GFP reporter cassette (Fig. 1) and tested these by injection into *Xenopus* embryos as described above. We first deleted the region P1D1 of BAC16 adjacent to BAC15. However, injection of BAC16-P1D1 or of constructs BAC16-P1D3 or BAC16-P1D4 with smaller deletions nested within that region recapitulated the GFP expression domains observed after injection of full length BAC16 suggesting that P1D1 does not contain any of its enhancers (Table 2).

We then deleted the other part of BAC16 (to the far side of BAC15). Neither this construct, BAC16-P2D1, nor the construct BAC16-P2D3 with a smaller deletion were able to recapitulate any of the GFP expression domains driven by BAC16 (Fig. 5C,C’; Table 2). However, all GFP domains driven by BAC16-FL were recovered after injection of a construct with the even smaller deletion P2D4 (Fig. 5D,D’; Table 2) indicating that all enhancers are located in the approximately 5.5 kbp domain deleted in P2D3 but not in P2D4 (BAC16P2D3-P2D4). In BAC16-P2D4 we also observed occasional expression in pharyngeal pouches (Fig. 5D,D’; Table 2), which was not seen in BAC16-FL or other BAC16 deletion constructs.

### Phylogenetic footprinting of genomic regions with enhancer activity

Using *X. tropicalis* as a reference genome in comparison with human, mouse, rat and chick genomes, we performed phylogenetic footprinting on the two genomic regions showing enhancer activity in our study. This analysis confirmed the presence of three conserved regions in BAC15P2D2-P2D3, which were identified in a previous study by Ishihara *et al*.^28^ as conserved noncoding regions CNS13, CNS14, CNS15 based on a mouse reference genome. However, in *Xenopus* these regions map to exons 8, 7 and 5, respectively of *Eya1* (Fig. 1). No conserved sequences mapped to the other region (BAC16P2D3-P2D4).

## Discussion

Our findings illustrate how a double BAC recombineering strategy with a reporter gene cassette (insert 1) and various deletion cassettes (insert 2) can identify cis-regulatory elements in large genomic regions. Applying this strategy to the *Eya1* locus in *X. tropicalis* where candidate enhancers are distributed over a 329 kbp wide genomic region, we identified two novel genomic regions with enhancer activity, one mapping to the *Eya1* locus itself and containing several of its introns (BAC15P2D2-P2D3), the other one mapping far upstream (135 kbp) of the first Eya1 exon (BAC16P2D3-P2D4). These regions are 9 and 5.5 kbp in length, respectively, and thus falls within the range that permits cloning into plasmid vectors and further analysis by classical “enhancer bashing”^40^. Such further analysis will be required to pinpoint individual enhancers within these regions and to establish which of the predicted transcription factor binding sites within these enhancers are in fact essential for enhancer activity.

While a previous study has shown that a subset of conserved noncoding sequences surrounding the *Eya1* locus serve as enhancers, none of these were located in these two regions^28^. Although one of our regions (BAC15P2D2-P2D3) contains three of the conserved noncoding sequence elements (CNS13, CNS14, CNS15) of Ishihara *et al*.^28^, none of them showed any enhancer activity after electroporation into chick embryos. In *Xenopus*, sequences corresponding to these three domains (with sizes ranging from 80–180 base pairs) actually correspond to exons 8, 7 and 5, respectively of *Eya1*. The fact that these sequences were classified as “conserved noncoding sequences” by Ishihara *et al*.^28^ based on a mouse reference genome suggests that they may represent exons that are facultatively included in some *Eya1* splice variants rather than cis-regulatory elements. Our second region (BAC16P2D3-P2D4) does not contain any of the CNS elements identified by Ishihara *et al*.^28^. This suggests that our two regions contain additional enhancers of Eya1 with relatively low sequence conservation among vertebrates. Taken together with previous studies that show that many enhancers undergo rapid sequence evolution^29,30^ this highlights the limitations of similarity based tools for enhancer prediction.

Conversely, however, Ishihara *et al*.^28^ identified a few *Eya1* enhancers in amniotes that map to parts of our BACs which do not show enhancer activity in *Xenopus*. Their CNS22 which directs reporter gene expression in the chick head ectoderm is located in region P1D2 of BAC15, in which we did not detect enhancer activity. Moreover, their CNS23 and CNS26, which regulate expression in the chick olfactory placodes (CNS23), otic placodes (CNS23, CNS26), somites (CNS26) and brain (CNS26) among other domains are located in region P1D1 of BAC16, in which we likewise did not detect enhancer activity. This raises the possibility that the phylogenetic sequence conservation of these CNSs may reflect other constraints than conserved enhancer activity, whereas the expression of Eya1 in particular domains may be regulated by different enhancers in different species.

The existence of multiple partly redundant enhancers driving expression in the same domain may facilitate such evolutionary flexibility of enhancer usage. In our study we found a surprising similarity in the enhancer activity of the two regions BAC15P2D2-P2D3 and BAC16P2D3-P2D4, each of which drives expression in the epidermis, PPR and somites even though there are no significant sequence similarities between the two regions. Multiple enhancers for similar expression domains were also found by Ishihara *et al*.^28^, including four enhancers for expression in the otic vesicle, four enhancers for expression in head ectoderm, four enhancers for trigeminal ganglion expression and two enhancers for somite expression. Such duplicate enhancers (with a proximal “primary” and a more remote “shadow” enhancer) have now been identified in many developmental genes of different animals and were proposed to promote robustness of gene expression in variable environmental and genetic contexts^41,42^.

However, there is an additional layer of complexity because many individual enhancers for Eya1 appear to drive expression in a combination of different domains, for example trigeminal ganglia, head ectoderm and somites (CNS28); trigeminal ganglia, otic vesicle and neural crest (CNS3); trigeminal ganglia, otic vesicle, brain and somites (CNS26); and otic vesicle and olfactory placode (CNS23)^28^. The two regions with enhancer activity identified here also drive expression in multiple domains, but our data lack sufficient resolution to determine whether this is due to the presence of multiple enhancers or of one or several multifunctional enhancers in each region.

Finally, our findings demonstrate that GFP reporter gene expression mediated by each of the two regions extends beyond endogenous domains of Eya1 expression and includes strong expression in the neural plate and in scattered epidermal cells. Some of the enhancers identified by Ishihara *et al*.^28^ also drove reporter gene expression in atypical domains, for example migrating neural crest cells or lateral plate mesoderm, where Eya1 is not expressed. Several different mechanisms may be involved here, but we currently lack sufficient information to determine which of those account for the extra expression domains observed. First, some of these domains may be experimental artefacts due to leaky GFP reporter expression even in the absence of enhancer activity. However, the fact that ectopic GFP expression domains are only observed for some of our BAC reporter constructs but not for others makes this relatively unlikely. Second, differences in mRNA or protein stability between the GFP reporter and Eya1 may result in atypical expression patterns, in particular for domains with very dynamic or transitory expression. This may, for example, account for relatively strong and persistent GFP expression in the neural plate compared to the transitory neural expression of Eya1^23^. Third, enhancer driven expression domains may also be modulated by silencer elements located elsewhere on the chromosome^43^. Reporter constructs which contain an enhancer but lack the silencers will then show broader and atypical expression domains than the endogenous gene. The many CNSs identified by Ishihara *et al*.^28^ which show sequence conservation but lack enhancer activity are candidates for such silencers but further experimental evidence is needed to test this hypothesis.

## Methods

### Animals

All animal experiments were performed in full accordance with Irish and European legislation, were approved by the NUI Galway Animal Care Research Ethics Committee (ACREC, 003/10) and were covered under the animal license (Cruelty to Animals Act, 1876) B100/4291 to G. Schlosser.

### Constructs

BAC clones in pECCBAC1 carrying a chloramphenicol resistance gene were obtained from a BAC library recently generated from genomic DNA of *X. tropicalis* ^34^. Using a BAC genome browser, we identified several BACs covering the region between the *Eya1* locus and its neighboring genes *MSCI* (5′) and *XKR9* (3′) (Suppl. Table 1, Suppl. Figure 1, all BAC information is available on Xenbase (http://www.xenbase.org/) in the *Xenopus tropicalis* Version 9.0 Genome browser; using the community track named xthr.out.parsed.ALLBAC.gff). Two of these BACs, BAC15 and BAC16, covering the 5′ end of the *Eya1* coding region and upstream sequences were selected for the present analysis (Fig. 1). The plasmid pIS-GATA2-GFP used to generate the first insert was kindly provided by Rob Grainger and was generated by replacing the Pax6 promoter in pIS-Pax6-GFP^39^ with a zebrafish GATA2 minimal promoter (from the basic enhancer test vector in a previous study^44^). The pSK-Kana-RpsL plasmid was obtained from Addgene (plasmid 20871).

### PCR and purification of inserts

Insert 1 containing a *GFP* reporter and ampicillin resistance cassette (*Amp*) was amplified by PCR (95 °C: 1′, 72 °C: 45″, 72 °C: 2.5′; 25 cycles) from pIS-GATA2-GFP using primers containing overhangs with restriction sites (for subsequent verification of successful recombineering) and 50 base pair homology arms to BAC15 or BAC16, respectively (Suppl. Tables 2 and 3). For BAC15, which contained the first coding exon of Eya1 with the start codon, we used the plasmid to generate a small (2.8 Kb) insert with a 5′ *GFP* cassette and a 3′ *Amp* gene. The insert did not include the GATA2 minimal promoter (Fig. 2) since the insert will be under control of the Eya1 promoter after recombineering. The forward homology arm H1 was designed to target this insert to the first coding exon of *Eya1* exactly after the AUG start codon, whereas the reverse homology arm H2 was designed to bind to a sequence after a 34 nucleotide gap from AUG, thus inserting *GFP* in frame while truncating *Eya1* (Suppl. Table 2). For BAC16, which did not contain the Eya1 coding region or Eya1 promoter we generated a larger (4.5 Kb) insert with a 5′ *Amp* gene and a 3′ cassette comprising the GATA2 minimal promoter and *GFP* (Fig. 2). Homology arms H1 and H2 were designed to target this insert into the center of the noncoding region of BAC16 (Suppl. Table 3).

Insert 2 containing a kanamycin resistance cassette was amplified by PCR (95 °C: 1′, 72 °C: 45″, 72 °C: 2.5′; 25 cycles) from pSK-Kana-RpsL using primers containing overhangs with restriction sites (for subsequent verification of successful recombineering) and 50 base pair homology arms to BAC15 or BAC16, respectively (Suppl. Tables 4 and 5). Homology arms were designed to flank regions of various sizes in the BACs (Fig. 2). Recombineering of insert 2 will thus create deletions of various sizes and replace them by the kanamycin resistant cassette leaving insert 1 in place to allow screening for GFP reporter expression. Primers for creating deletion constructs in BAC15 and 16 are listed in Suppl. Figure 4 and 5, respectively.

After PCR amplification, 2 μl of DpnI (37 °C, 2 h) was added to the PCR reaction to digest the template plasmids. The insert was purified by running the entire reaction on a 0.5% agarose gel and cutting out the proper bands followed by gel extraction (Qiagen, Cat. No. −28706). Complete restriction digestion of the template was confirmed by running 2 μl of the gel extract on a 1.2% agarose gel.

### BAC recombineering

BAC15 and 16 were obtained as stabs of DH10B bacteria which were plated on agar plates containing chloramphenicol (25 µg/ml) overnight. 100 ml Luria-Bertani medium (LB) containing chloramphenicol was inoculated with a single colony and grown overnight at 32 °C. BACs were extracted using the NucleoBond Kit (Macherey-Nagel, Cat.no. 740579) and resuspended in nuclease free water. Next BACs were electroporated (2 µl for 50 µl bacteria; 25µF, 2.5 kV, 25 Ohm, 2 millisecond) into bacterial strains SW102 or EL250 containing the λ prophage based recombineering system using previously established protocols (Warming *et al*., 2005; Wang *et al*., 2009). To select for successful transformants, bacteria were plated on agar plates containing chloramphenicol (25 µg/ml) overnight. Insert 1 was then electroporated into SW102 or EL250 cells containing the BACs and recombineering induced by a 42 °C heat shock which inactivates the λ repressor^45,46^. Uninduced control bacteria were kept at 32 °C, a temperature at which recombineering should be repressed by active λ repressor. To select for bacteria containing recombineered BACs, bacteria were plated on agar plates containing chloramphenicol (12.5 µg/ml) and ampicillin (100 μg/ml) overnight. To create deletion constructs of BACs containing insert1, SW102 or EL250 cells containing the BACs were subjected to a second round of recombineering using insert 2 (Fig. 3C). To select for bacteria containing double recombineered BACs, bacteria were plated on agar plates containing chloramphenicol (25 µg/ml), ampicillin (100 μg/ml) and kanamycin (100 μg/ml) overnight. BACs were extracted using the NucleoBond Kit (Macherey-Nagel, Cat.no. 740579)

PCR (95 °C: 1′, 72 °C: 45″, 72 °C: 2.5′; 17 cycles) was used to verify successful recombineering of inserts 1 and 2 using the BAC as a template and forward (FB) and reverse primers (RB) complementary to BAC sequences immediately flanking the region of insertion (Fig. 3A; Suppl. Tables 2–5). In addition, restriction digestion of BACs with enzymes recognizing the primer-specific restriction sites followed by agarose gel (1.2%) electrophoresis was sometimes used to confirm recombineering. The correct orientation of insert 1 after recombineering was confirmed by PCR using the BAC as a template and a pair of forward and reverse primers with one member of the pair (forward: FI; reverse: RI) complementary to sequences of the insert (*GFP* or *Amp*) and the other member (reverse: RB; forward: FB) complementary to a BAC sequence immediately flanking the region of insertion (Fig. 3A, Suppl. Tables 2 and 3). Finally proper insertion and integrity of the insert was verified by sequencing using FB,RB, FI and RI as sequencing primers.

### Microinjection

Embryos of *Xenopus laevis* were obtained by hormone induced egg laying followed by *in vitro* fertilization, staged according to (Nieuwkoop and Faber, 1967)^47^ and injected according to standard procedures^48^. 5 nl of a 100 ng/µl BAC solution were injected into one-cell stage embryos. After survival into neural plate or tailbud stages, embryos were live imaged for GFP fluorescence using an Olympus SZX16 stereomicroscope equipped with a 460–490 GFP filter set and a DP71 camera and subsequently fixed in 4% PFA.

### In situ hybridization

Wholemount *in situ* hybridization was carried out under high stringency conditions at 60 °C as previously described^16^ using a digoxigenin-labeled antisense RNA probe against *GFP* generated by *in vitro* translation from pCMTEGFP (kindly provided by Doris Wedlich).

### Phylogenetic footprinting

For phylogenetic footprinting *X. tropicalis* was used as a reference genome and was compared with human, mouse, rat and chick genomes using ECR browser (http://ecrbrowser.dcode.org), VISTA browser (http://genome.lbl.gov/vista/index.shtml) and Pipmaker (http://pipmaker.bx.psu.edu/pipmaker/).

### Data availability statement

All relevant data analyzed during this study are included in this published article.

## Electronic supplementary material

Supplemental material
