# Supplementary material for: Identification of novel cis-regulatory elements of Eya1 in Xenopus laevis using BAC recombineering
Source: Sci Rep. 2017 Nov 3;7:15033. doi: 10.1038/s41598-017-15153-7 (PMC5670250; doi:10.1038/s41598-017-15153-7)
Supplement: Supplementary file 1 — Supplemental material [file 41598_2017_15153_MOESM1_ESM.pdf]

Identification of novel cis-regulatory elements of *Eya1* in *Xenopus laevis* using BAC recombineering

Santosh Kumar Maharana, Nicolas Pollet, Gerhard Schlosser

Supplemental material

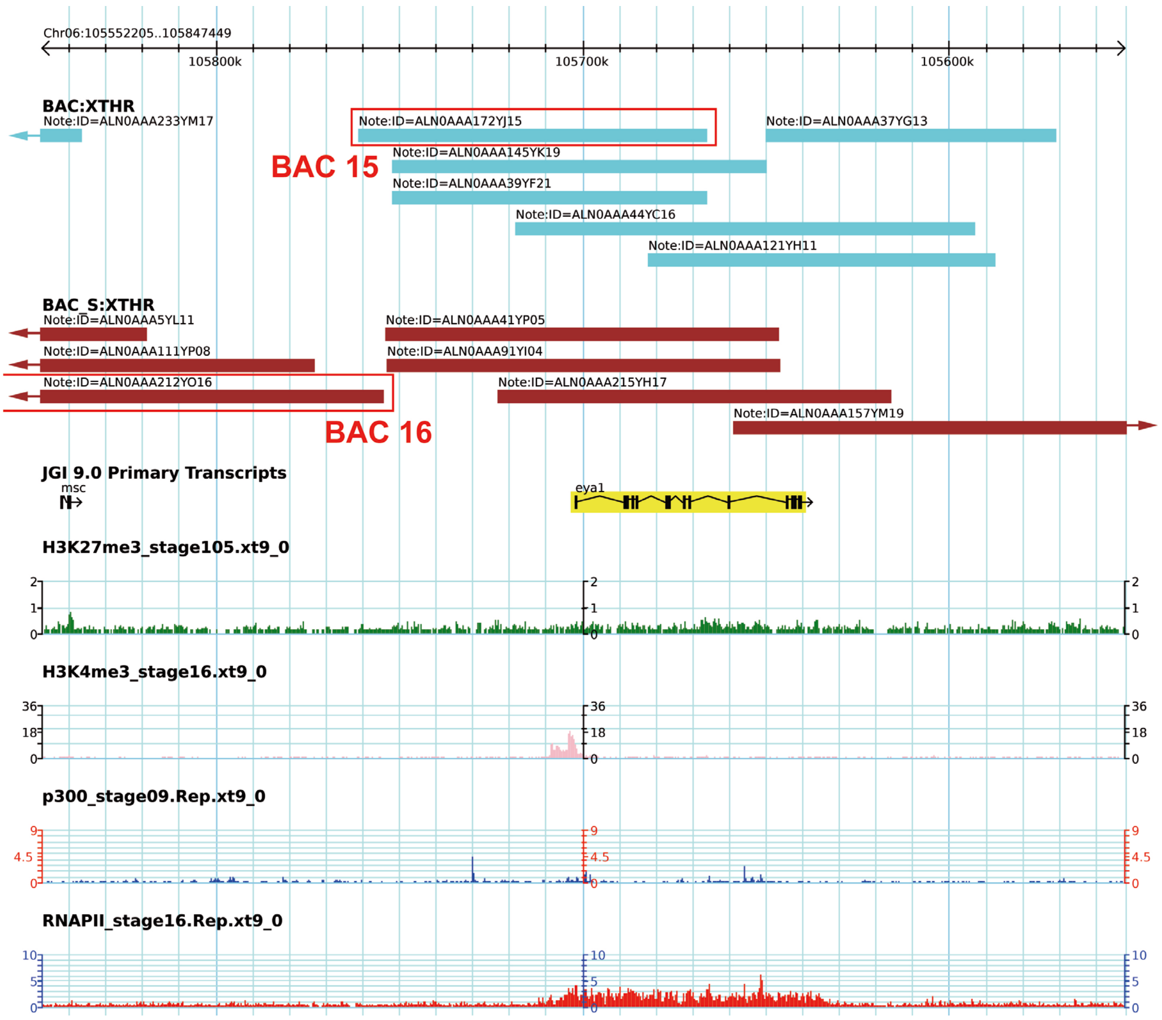

Suppl. Fig. 1. Alignment of various BACs with the *Eya1* genomic region in *X. tropicalis*. Screenshot from *Xenopus tropicalis* Version 9.0 Genome browser (http://www.xenbase.org/) using the community track named xthr.out.parsed.ALLBAC.gff). The position of various BACs from a *X. tropicalis* BAC library[34] relative to *Eya1* is indicated and the two BACs analyzed here, termed BAC15 and BAC16, are highlighted. Note that the precise position of the plus end (right side) has not been reported for BAC16 (see Suppl. Table 1).

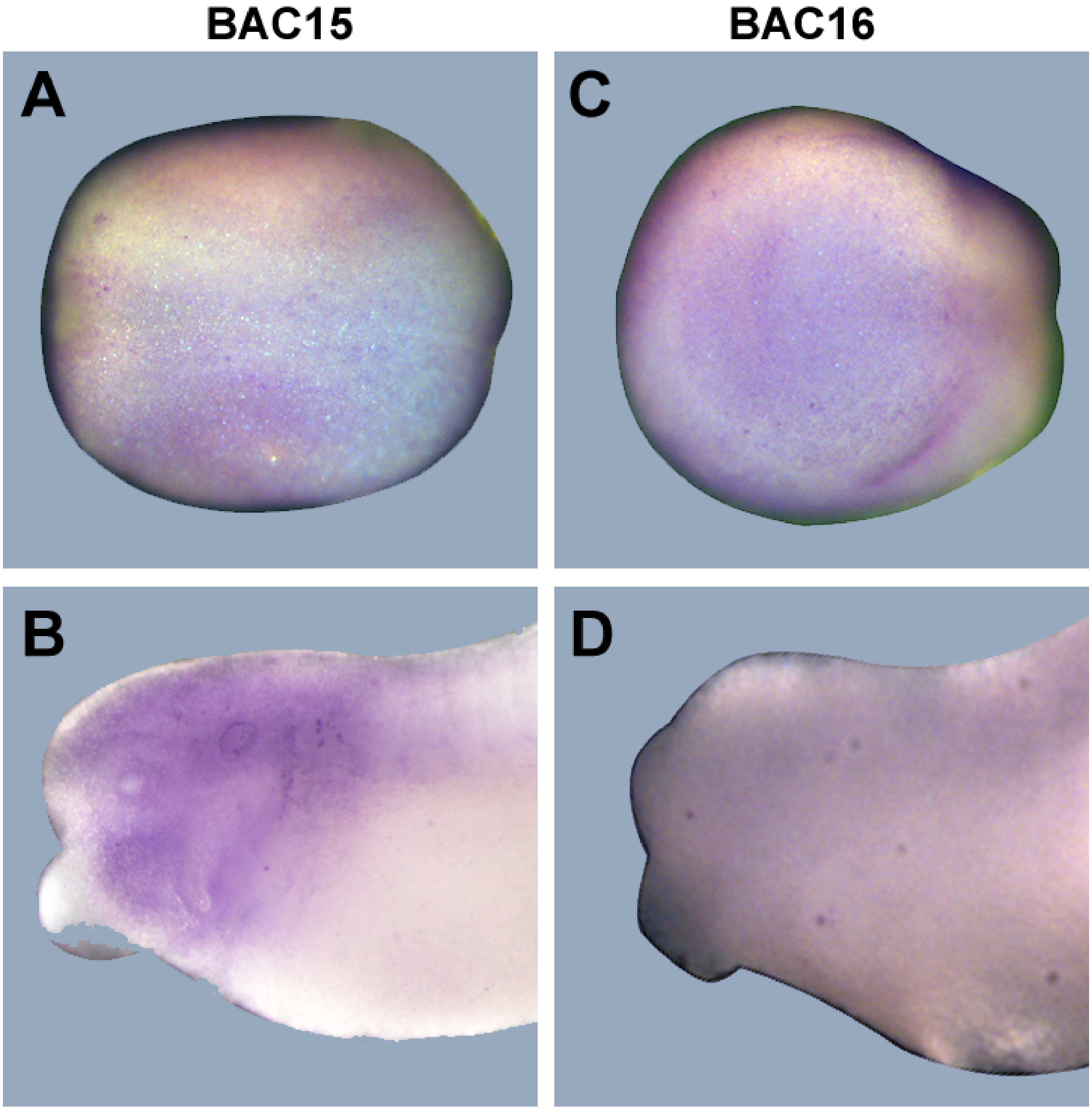

Suppl. Fig. 2. Control embryos injected with BACs without reporter cassettes. Embryos of *Xenopus laevis* at neural plate (A, C: dorsal view, anterior to the left) and tailbud stages (B, D: lateral view, anterior to the left) after injection with BAC15 (**A,B**) or BAC16 (**C,D**) constructs at the one-cell stage. In the absence of reporter cassettes, no expression domains are observed after in situ hybridization for *GFP*.

Suppl. Table 1

BACs covering the *Eya1* coding region and its vicinity

BAC Identifier	Size (bp)	End Seq Genbank	End Seq orientation	Chr. 06 map[1]	Name used here
ALN0AAA37YG13	79 030	JY026638	minus	105 650 055	BAC13
		JS989568	plus	105 571 025	
ALN0AAA44YC16	125 252	JY073067	minus	105 718 400	BAC16A
		JY014952	plus	105 593 148	
ALN0AAA111YP08	94 084	JS920919	minus	105 880 209	BAC08
		JS970870	plus	105 786 125	
ALN0AAA145YK19	101 712	JY006731	minus	105 751 764	BAC19
		JY006221	plus	105 650 052	
ALN0AAA172YJ15	94 591	JS939121	minus	105 760 926	BAC15
		JS973010	plus	105 666 335	
ALN0AAA215YH17	131 335	JS946481	minus	105 747 150	BAC17
		JY043529	plus	105 615 815	
ALN0AAA212YO16	81 285	JS943664	minus	105 862 090	BAC16
		JS994048	plus	<105 763 626[2]	

[1]Chr. 06 map is based on *X. tropicalis* genome assembly v9.0 available on Xenbase
[2]Precise position of plus end has not been reported but has to be outside of outer homology arm for our BAC16P1D1 construct which starts at position 105 763 626.

Suppl. Table 2

Primers for recombineering of insert 1 (reporter) into BAC 15

Primer Name[1]	Primer Sequences (5' – 3') (black: BAC sequence; red: restriction site; blue: insert sequence)
**Xt_Eya1_BAC15_GFP_H1_AscI_F**	CGATGGCTGGAGGGTTACACGCTCTGAAATGCACTTCTCTTA TTTCTAGGTGGCGCGCCTCTAAGCGCGGACACTTGAG
**Xt_Eya1_BAC15_Amp_H2_AsiSI_R**	AATTATCAAGGCCTGTCCCACTGGGAGAATCACCACTGCCAC TAAGTCTACGCGATCGCCGCTCAGTGGAACGAAAACT
**Xt_Eya1_BAC15_FB**	ACAGGATATGGTCAGTTTGTATTTTTG
**Xt_Eya1_BAC15_RB**	ACCCAAGAACAAACACTGTCTT
**Xt_Eya1_Amp_BAC15_FI**	GCGGTATCATTGCAGCACTG
**Xt_Eya1_GFP_BAC15_RI**	TAGGTCAGGGTGGTCACGA

[1]F: Forward primer for BAC recombineering;
R: Reverse primer for BAC recombineering;
FB: Forward primer binding to BAC adjacent to insertion for verification of insertion;
RB: Reverse primer binding to BAC adjacent to insertion for verification of insertion;
FI: Forward primer binding to insert for verification of insert orientation;
RI: Reverse primer binding to insert for verification of insert orientation

Suppl. Table 3

Primers for recombineering of insert 1 (reporter) into BAC 16

Primer Name[1]	Primer Sequences (5' – 3') (black: BAC sequence; red: restriction site; blue: insert sequence)
**Xt_Eya1_BAC16_Amp_H1_AscI_F**	TTGGTTGTTTAAAGCACAGTTGACATAATATTGGTGTGTAACC CTCCAGTGGCGCGCCGGCACTTTTCGGGGAAATGT
**Xt_Eya1_BAC16_GFP_H2_AsiSI_R**	TATATTGTCATGAGGAAGGAAATATGTGTCACATTGCAGTCTA CATTAACGCGATCGCTTCAGGTTCAGGGGGAGGTG
**Xt_Eya1_BAC16_FB**	GGACTTGCCTGAGATGCTGA
**Xt_Eya1_BAC16_RB**	GTGGTTCTCCCAGAGAGCAA
**Xt_Eya1_GFP_BAC16_FI**	ATGGCCGACAAGCAGAAGAA
**Xt_Eya1_Amp_BAC16_RI**	TAATACCGCGCCACATAGCA

[1]F: Forward primer for BAC recombineering;
R: Reverse primer for BAC recombineering;
FB: Forward primer binding to BAC adjacent to insertion for verification of insertion;
RB: Reverse primer binding to BAC adjacent to insertion for verification of insertion;
FI: Forward primer binding to insert for verification of insert orientation;
RI: Reverse primer binding to insert for verification of insert orientation

Suppl. Table 4

Primers for recombineering of insert 2 (deletion) into BAC15

Primer Name[1]	Primer Sequences (5' – 3') (black: BAC sequence; blue: insert sequence)
**Xt_Eya1_BAC15_P1H1_F**	AACTTAACAGCTGGGAAAGAAAAATCACTCTCAAAGTTGT CTAAAATGTAGCTTGCAGTGGGCTTACATG
**Xt_Eya1_BAC15_P1H2_R**	ATAGAAGTTAATAAAAAAGCAAGATCCTATATTATCATTAG CATAAGACACGGCCGCTCTAGAACTAGTG
**Xt_Eya1_BAC15_P1H3_R**	AATAAGTGTCCGATAAATAATTAATTTTAATCTCATTATAGA CTTTTTCACGGCCGCTCTAGAACTAGTG
**Xt_Eya1_BAC15_P1H4_R**	CATTTTTACTTCAAGTCTCCCACCAGAACCGTACACTCTCC ATACGGAACCGGCCGCTCTAGAACTAGTG
**Xt_Eya1_BAC15_P1H5_R**	CTGAGACGTTTACAGCAATGAAAAACTGGTTAATTGAAAT CCATAAAGCTCGGCCGCTCTAGAACTAGTG
**Xt_Eya1_BAC15_P1H1_FB**	ACAGCTGGGAAAGAAAAATCACT
**Xt_Eya1_BAC15_P1H2_RB**	ACAGCTGGGAAAGAAAAATCACT
**Xt_Eya1_BAC15_P1H3_RB**	TGGCATTGCAAACATTCCTGG
**Xt_Eya1_BAC15_P1H4_RB**	GTTTTACCAACGCAGGGCAA
**Xt_Eya1_BAC15_P1H5_RB**	ACCATGAAAAGCAAAACATTGGT
**Xt_Eya1_BAC15_KanaP1H1_RI**	CACGGCATACTTTACGCAGC
**Xt_Eya1_BAC15_KanaP1H2_RI**	CGGACCGCTATCAGGACATAG
**Xt_Eya1_BAC15_P2H1_F**	CACATAAACAGCAATTCCATGACACCAAATGGCACAGAAG GTGAGTCCCAAGCTTGCAGTGGGCTTACATG
**Xt_Eya1_BAC15_P2H2_R**	GGGAAATGTTTTCAGAGAAAAGGGATAACTAACTCAAGCA CATAGCATGTCGGCCGCTCTAGAACTAGTG
**Xt_Eya1_BAC15_P2H3_R**	GCTATAACATTGTATTGAGCCTTTACACATTCTCATAAAGA GTTTGTTCACGGCCGCTCTAGAACTAGTG
**Xt_Eya1_BAC15_P2H4_R**	CAACGCGCCATAAATTAATTAGCAACTACATATATGATGG CTTCCCTGTGCGGCCGCTCTAGAACTAGTG
**Xt_Eya1_BAC15_P2H5_R**	AATTTGCTAAGCAACCAGCCCACAGTGTTCACTAGACTCA TGCTGTGCCCCGGCCGCTCTAGAACTAGTG
**Xt_Eya1_BAC15_P2H1_FB**	CACAGGATATGGTCAGTTTGTATTTTT
**Xt_Eya1_BAC15_P2H2_RB**	GGGAAATGTTTTCAGAGAAAAGGGA
**Xt_Eya1_BAC15_P2H3_RB**	AGTAAGATGAAGTGTATGCAAAGGTG
**Xt_Eya1_BAC15_P2H4_RB**	GTTGGCAACCTTACACCCTT
**Xt_Eya1_BAC15_P2H5_RB**	AGCGCCGACTATACCTGATG

[1]F: Forward primer for BAC recombineering;
R: Reverse primer for BAC recombineering;
FB: Forward primer binding to BAC adjacent to insertion for verification of insertion;
RB: Reverse primer binding to BAC adjacent to insertion for verification of insertion;
FI: Forward primer binding to insert for verification of insert orientation;
RI: Reverse primer binding to insert for verification of insert orientation

Suppl. Table 5

Primers for recombineering of insert 2 (deletion) into BAC16

Primer Name[1]	Primer Sequences (5' – 3') (black: BAC sequence; blue: insert sequence)
**Xt_Eya1_BAC16_P1H3_F**	CGAATTAAAAAAAGTTGTGTGCATCAATAAAAAATGGTT AATGATAGAATAGCTTGCAGTGGGCTTACATG
**Xt_Eya1_BAC16_P1H4_R**	AGGGCAGCACTCCTAGGATTAAAGAGAAAATCGTAAA CTAAAAGATAAAACGGCCGCTCTAGAACTAGTG
**Xt_Eya1_BAC16_P1H5_R**	ATTTGTACAAGGTAATCTTGGAATTACTCTGTAAGCAT GTCCATAATGGCTCGGCCGCTCTAGAACTAGTG
**Xt_Eya1_BAC16_P1H6_R**	TACACCTGAAAGTCCACCTTGAGGGTCAGTTAGGATG AGATTTGTGAAATACGGCCGCTCTAGAACTAGTG
**Xt_Eya1_BAC16_P1H7_R**	TCGTGACTCGTGACTGACTATTGTTTTTTTGTCCATCA GGAAACCATTGTCGGCCGCTCTAGAACTAGTG
**Xt_Eya1_BAC16_P1H3_FB**	ACTGTCATCCTGTGCAAAGGT
**Xt_Eya1_BAC16_P1H4_RB**	AGGGCAGCACTCCTAGGAT
**Xt_Eya1_BAC16_P1H5_RB**	AAAAAGGGGGCTCAGGGAAA
**Xt_Eya1_BAC16_P1H6_RB**	TCATTTGGCATTCGTATCTTTAGAGA
**Xt_Eya1_BAC16_P1H7_RB**	ATGGGAGACTTTCCTTGGGC
**Xt_Eya1_BAC16_P2H1_F**	AATAAAACCAGGACATTCATAGGAAAGTGTGGCCATTT GGGATTGCCCTTAGCTTGCAGTGGGCTTACATG
**Xt_Eya1_BAC16_P2H2_R**	AAGGTCGCAGGCATGTAAAAAAAGTTGCAGTTGTGTCA AAAAAGTTACGGCGGCCGCTCTAGAACTAGTG
**Xt_Eya1_BAC16_P2H3_R**	TTCTGCAGCCCGCCTCCCTCAACTTGCTTCATTGAATC CCTGTATAAGACCGGCCGCTCTAGAACTAGTG
**Xt_Eya1_BAC16_P2H4_R**	AAGTTTGGGTCACTATAGCATAAGTATCTTTAACCATG AGAACTGTGTTCCGGCCGCTCTAGAACTAGTG
**Xt_Eya1_BAC16_P2H5_R**	TACTCTGTATGTCCAATGTATGAAACCCACTTATTGTAC AGCGCTGCGGACGGCCGCTCTAGAACTAGTG
**Xt_Eya1_BAC16_P2H1_RB**	CTCAAGGGGCTGAGAAGTGA
**Xt_Eya1_BAC16_P2H2_FB**	AAAGGTTGGGGATCCCTATGC
**Xt_Eya1_BAC16_P2H3_FB**	TCACAACTCTGGTCATGCCC
**Xt_Eya1_BAC16_P2H4_FB**	CTGCCCTCAGTGTAGCCAA
**Xt_Eya1_BAC16_P2H5_FB**	CCAAGGTAATATAAGAGCTCTCAGC

[1]F: Forward primer for BAC recombineering;
R: Reverse primer for BAC recombineering;
FB: Forward primer binding to BAC adjacent to insertion for verification of insertion;
RB: Reverse primer binding to BAC adjacent to insertion for verification of insertion;
